# Morphology and properties of foamed high crystallinity PEEK prepared by high temperature thermally induced phase separation

**DOI:** 10.1002/app.51423

**Published:** 2021-07-26

**Authors:** Dmitrii Rusakov, Angelika Menner, Florian Spieckermann, Harald Wilhelm, Alexander Bismarck

**Affiliations:** ^1^ Institute of Material Chemistry and Research, Polymer and Composite Engineering (PaCE) Group, Faculty of Chemistry University of Vienna Vienna Austria; ^2^ Materials Physics, Department Materials Science University of Leoben Leoben Austria; ^3^ Laboratory of Polymer Engineering (LKT‐TGM) Vienna Austria; ^4^ Department of Chemical Engineering Imperial College London London UK

**Keywords:** Poly ether ether ketone (PEE)K, porous polymers, Temperature induced phase separation (TIPS)

## Abstract

Polyetheretherketone (PEEK) is a high‐performance semi‐crystalline thermoplastic polymer with outstanding mechanical properties, high thermal stability, resistance to most common solvents, and good biocompatibility. A high temperature thermally induced phase separation technique was used to produce PEEK foams with controlled foam density from PEEK in 4‐phenylphenol (4PPH) solutions. Physical and mechanical properties, foam and bulk density, surface area, and pore morphology of foamed PEEK were characterized and the role of PEEK concentration and cooling rate was investigated. Porous PEEK with densities ranging from 110 to 360 kg/m^3^ with elastic moduli and crush strength ranging from 13 to 125 MPa and 0.8 to 7 MPa, respectively, was produced.

## Graphical abstract

Temperature induced phase separation of PEEK from 4‐phenylphenol solutions allows for production of high porosity PEEK foams with excellent mechanical properties.

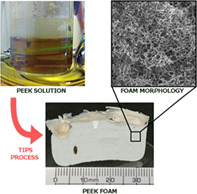

## 1 INTRODUCTION

Polymers are currently among the most commonly used materials globally because they are lightweight and durable, have good mechanical properties and low production costs. These features facilitate their use in a wide range of applications, such as consumer products, construction materials, insulation, films, membranes, and so on. One of the most promising classes of industrial high‐performance polymers are polyaryletherketones, which possess high continuous service temperatures, that is, the maximum temperature at which the polymers can be used without significant change in properties, outstanding mechanical properties and high chemical resistance.^1^, ^2^ One such high‐performance polymer is polyetheretherketone (PEEK) – a semi‐crystalline linear polymer with a high elastic modulus (E = 3–4 GPa) and tensile strength (σ ≈ 100 MPa) and a glass transition temperature (*T*_g_) of 143°C and melting temperature *T*_m_ of 343°C. The aromatic rings and saturated bonds within PEEKs structure provide good biocompatibility, high oxidation stability and chemical resistance at temperatures below *T*_g._ ^3^ In fact, at temperatures below *T*_g_ PEEK can only be dissolved in concentrated sulfuric and methanesulphonic acid.^4^ While at temperatures exceeding the glass transition temperature, it is possible to dissolve PEEK in high boiling solvents, such as dichloroacetic acid, benzophenone, 4‐phenylphenol, diphenyl sulfone, and chlorophenols.^5^, ^6^, ^7^

PEEK adheres well to carbon fibers,^8^ carbon nanotubes^9^ and other fillers,^10^ which can be used to further improve the mechanical and thermal properties of PEEK. PEEK and reinforced PEEK are widely used in biomedical applications as artificial scaffolds^11^ or as implants without requiring any additional coatings or modifications because of their bioinert nature.^12^, ^13^ PEEK is also used for the fabrication of membranes^14^ or high‐pressure fittings^15^ and construction materials for aerospace applications.^16^ More recently, PEEK found also use as material for additive manufacturing using fused deposition modeling.^17^

Porous polymers are a class of polymer materials, which combine low density with good mechanical properties and high surface areas.^18^ Porous polymers are consequently used as membranes,^19^ filtration materials,^20^ separators in batteries^4^ and scaffolds in biomedical applications.^21^ Foam density, pore morphology and pore size distribution all significantly influence the material properties of porous polymers, however, control of these properties for semicrystalline polymers during manufacturing is still challenging. Unfortunately, the insolubility of PEEK in many common solvents and its small melt processing window and low melt strength^22^ make it difficult to produce porous PEEK, for instance by chemical or physical blowing.^23^, ^24^ As PEEK is synthesized by step‐growth polymerization in polar high boiling point aprotic solvents at relatively high‐temperatures^15^ it is also difficult to produce PEEK foams directly during synthesis.

Despite these challenges, several methods to produce porous PEEK have been reported. One of the most common methods is the use of sacrificial porogens, such as sodium chloride^23^ or titanium wires,^25^ which are then removed by leaching, or by blowing (for instance using Clariant XH907 with azodicarbonamide as the main component).^24^ In porogen leaching, PEEK powder and spherical salt beads or other porogens are mechanically mixed, compression molded and sintered at high pressure and temperature (around or above melting temperature). The porogen is then washed out using water (in case of NaCl) or other specific leaching agents resulting in PEEK foams. However, the complete removal of porogens and production of mesoscale porosity is typically not straight forward. Porous PEEK can also be produced by laser sintering.^26^ PEEK foams were produced by physical blowing, that is, by absorbing supercritical CO_2_ at high pressure in PEEK followed by expansion of the absorbed CO_2_ at temperatures ranging from 240 to 340°C.^27^ CO_2_ was also used as a foaming agent to produce foamed PEEK/polyetherimide (PEI) blends^28^; PEEK/PEI blends were saturated with CO_2_ at temperatures (close or above *T*_g_) followed by expansion to produce the foamed material. The main disadvantages of gas foaming are lack of control of the pore morphology and the tendency of gas bubbles to coalescence because of PEEK's low melt strength.^22^ Emulsion templating can also be used to produce high porosity high‐performance polymer foams (for instance from polyvinylidene fluoride (PVDF), polytetrafluoroethylene (PTFE) and PEEK), however, significant shrinkage of the materials occurs during the final sintering step.^29^ More recently, Talley et al^30^, ^31^, ^32^ described a method to produce porous PEEK aero/xerogels with a foam density of around 0.3 g/cm^3^ by dissolving PEEK in high boiling solvents (dichloroacetic acid and 4‐chlorophenol) followed by precipitating PEEK at temperatures above *T*_g_.

We recently showed that low density (~0.36 g/cm^3^) monolithic PEEK, polyetherketoneketone (PEKK), and PEI foams with very high E‐moduli (97 MPa) can be produced using high temperature thermally‐induced phase separation (HT‐TIPS).^33^ The TIPS method is based on the temperature dependent solubility of polymers in specific solvents. The polymer of interest is dissolved under active mixing until a transparent solution is obtained. Then, the solution is cooled at a specific cooling rate, until liquid–liquid or liquid–solid phase demixing occurs forming a polymer‐rich, solvent‐poor and a polymer‐poor, solvent‐rich phase. Subsequent removal of the solvent yields porous polymers.

The research reported herein builds from our previous work, with particular focus on the production of porous PEEK by broadening the scope of polymer/solvent concentrations and cooling rates used in its formation. We will show that it is possible to tailor the foam density and morphology, and thus the mechanical properties of porous PEEK over a wider range than achieved by Talley et al.^30^, ^31^, ^32^ Most importantly, the mechanical properties of the PEEK foams reported here significantly exceed those reported previously. We will also provide evidence that these improved mechanical properties correlated well with high degrees of crystallinity in our PEEK foams, which are achieved using our HT‐TIPS method.

## 2 EXPERIMENTAL SECTION

### 2.1 Materials

PEEK APC‐2 powder with average particle size (SMD or Sauter mean diameter) = 145 μm (see Figure S1 in SI) was kindly supplied by Cytec (Solvay Group). 4‐Phenylphenol (melting temperature *T*_f_ ~ 166°C, boiling point *T*_b_ ~ 321°C) with a purity of 97% was purchased from Sigma‐Aldrich and was used as solvent for PEEK. Iron(III) chloride (anhydrous for synthesis) was purchased from Sigma‐Aldrich. Ethanol with a purity of 96% (Brenntag NV, Belgium) was used for purification of porous PEEK. Acetone with 99.5% purity was supplied by Thermo Fisher Scientific. All materials were used as received.

### 2.2 Preparation of porous PEEK

**FIGURE 1:**
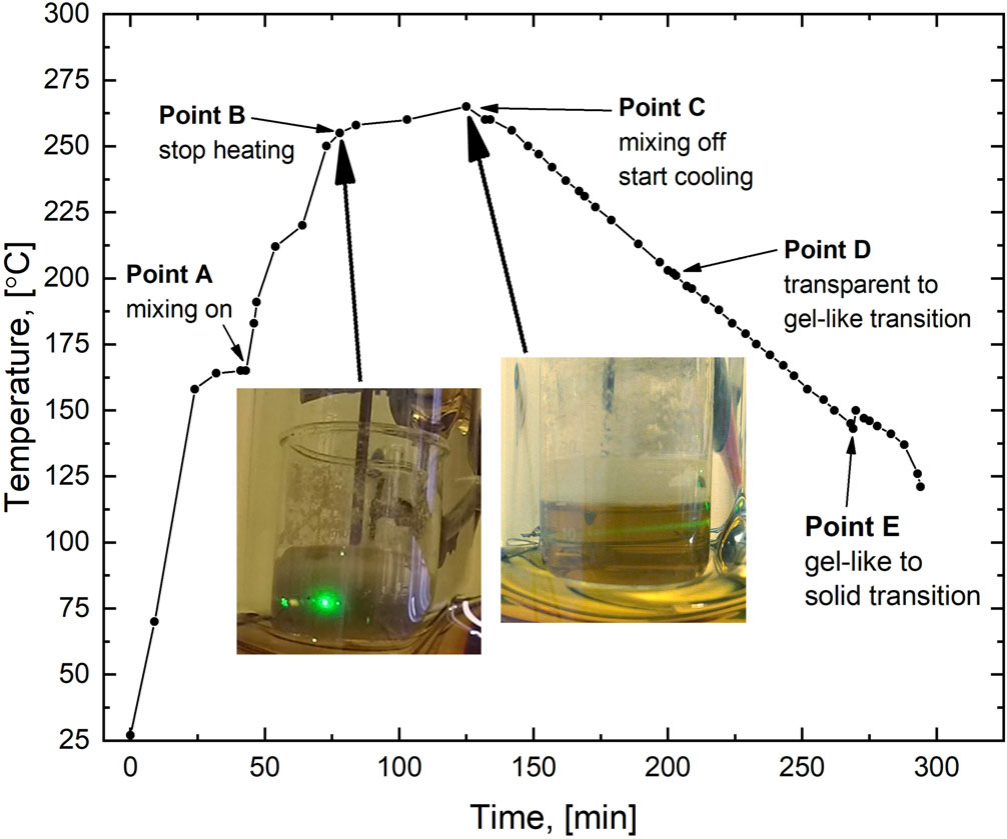
Characteristic heating/cooling profile for sample **D2**. The insert shows how we determined the solution state using a green laser pointer. Insert pictures can also be found in **SI** **Figure S2** [Color figure can be viewed at wileyonlinelibrary.com]

Various amounts of PEEK and 4PPH powder were weighed (see Table 1 for formulations and process parameters), and mechanically mixed in a 50 ml borosilicate glass beaker to produce a homogenous mixture of the two powders (PEEK and 4PPH), before a glass‐coated magnetic stir bar was added. The beaker containing the blend was then placed into a 250 ml double‐walled, round‐bottom borosilicate glass vessel (GlasKeller Basel AG, CH) with a two necked glass cap (DN 60), which was connected to a high‐temperature thermostat with an active cooling system (Huber Unistat T305W HT, D) with a maximum operating temperature of 300°C using insulated high‐temperature resistant hoses. A calibrated precision core thermometer (Erbo TFX 410, Ingolstadt, D) was used to control the temperature inside the PEEK/4PPH mixture. PEEK was dissolved in 4PPH by increasing the vessel temperature until the 4PPH melted (~170–180°C, Figure 1 point **A**), at which point stirring commenced. The temperature was further increased until the dissolution temperature (Table 1). Stirring continued for 15 to 25 min until a clear solution was observed (Figure 1, point **C**). The clear solution point was determined using a green laser pointer (see insert in Figure 1). This temperature depended on the PEEK concentration. It was impossible to produce a homogeneous solution when trying to dissolve more than 20 wt% PEEK in 4PPH. After confirming the clear solution point, stirring was stopped and the solution cooled at set cooling rates β (Figure 1 from **C**, and Table 1). During cooling, the solution started to become turbid and then gel‐like (Figure 1 **D**) in a relatively narrow temperature range (Table 1). After solvent crystallization, observable by the temperature increase recorded due to the release of latent heat of crystallization of 4PPH (Figure 1, **E**), the vessel temperature was further decreased at the same cooling rate until 120–110°C (inside the samples) to ensure a homogeneous temperature profile of the polymer‐solvent mixture. Finally, the samples were removed from the beaker and the crystalline solvent removed by Soxhlet extraction using ethanol for 48 h before being dried until constant weight in a vacuum oven at 60°C for 24 h. At least 5 repeats were performed for porous PEEK samples which were structurally sound to ensure reproducibility.

**TABLE 1:** *HT‐TIPS process parameters (heating and cooling rates) used for the production of foamed PEEK samples: Cooling rate,* β; temperature, T @ B, at which temperature was kept constant till point C was reached while mixing continued (point B); mixing time, t_B‐C_, at constant temperature required to produce a transparent PEEK solution (see Figure 1); temperature, *T*_gel_ @ D, where PEEK solution started to become cludy exhibiting a gel‐like character; and solvent (4PPH) crystallization temperature, *T*_f_ @ E

Sample name	[PEEK]/wt%	β/[°C/min]	T @ B/[°C]	*t*_B‐C_/[min]	*T*_gel_ @ D/[°C]	*T*_f_ @ E/[°C]
**A1**	5	0.5	248 ± 4	15	—	146 ± 1
**A2**	5	1	248 ± 3	15	—	147 ± 1
**A3**	5	2	249 ± 1	15	—	151 ± 1
**A4**	5	10	248 ± 4	15	—	150 ± 1
**B1**	10	0.5	262 ± 8	20	172 ± 4	143 ± 1
**B2**	10	1	262 ± 4	20	166 ± 3	146 ± 1
**B3**	10	2	256 ± 3	20	160 ± 2	148 ± 2
**B4**	10	10	259 ± 2	20	—	150 ± 3
**C1**	15	0.5	259 ± 4	25	195 ± 5	149 ± 6
**C2**	15	1	261 ± 3	25	181 ± 9	148 ± 5
**C3**	15	2	259 ± 1	25	181 ± 1	145 ± 4
**C4**	15	10	258 ± 1	25	—	150 ± 1
**D1**	20	0.5	264 ± 3	30	200 ± 4	142 ± 2
**D2**	20	1	261 ± 1	30	197 ± 8	143 ± 1
**D3**	20	2	260 ± 3	30	199 ± 7	145 ± 7
**D4**	20	10	258 ± 3	30	Not visible	151 ± 1

### 2.3 Characterization of porous PEEK

**Pore structure**: A benchtop scanning electron microscope (JEOL JCM‐6000, JEOL Ltd., Japan) was used to investigate the pore morphology of porous PEEK. Pieces of less than 1 cm in longitudinal direction and 1–2 mm in thickness were broken off from the porous PEEK samples and fixed to the sample holder using conductive carbon stickers. Silver paint (Achseon 1415, G 302, Agar Scientific, UK) was applied around the sample edge to increase conductivity prior to gold coating the samples in an argon atmosphere using a fine coater (JEOL JFC‐1200, JEOL Ltd, Japan). SEM micrographs were collected at various magnifications from numerous sites on each sample to characterize the pore structure.

**Absolute (skeletal) density** $\rho_{s}$ was measured using a helium gas‐displacement pycnometer (AccuPyc II 1340, Micromeritics Ltd, Norcross). Porous PEEK samples were cut (for 5 wt% samples) or broken (for 10, 15, and 20 wt% samples) into irregularly shaped pieces (each side of the piece was from 5 to 10 mm) from various positions of the sample, with a total mass of 0.3–0.4 g, then, weighed, and placed into a 3.5 cm^3^ measurement chamber and its volume determined.

**Bulk (foam) density** $\rho_{f}$ was determined using an envelope density analyzer (GeoPyc 1360, Micromeritics Ltd., Norcross). Three pieces of each porous PEEK sample approx. 10 × 10 × 10 mm^3^ in size were cut from the porous PEEK for individual measurements. The porosity P was calculated using as follows:

(1) $$P=\left(1-\frac{\rho_{f}}{\rho_{s}}\right)$$

**Mechanical properties** were measured in compression adapting the ISO 844:2001 standard using a dual column universal test frame (Instron 5969, Darmstadt, Germany) equipped with a 50 kN load cell. Porous PEEK samples were cut from their original cylindrical shape using a band saw (PROXXON MBS 240/E) into cubes with dimensions of 10 × 10 × 10 mm^3^. After cutting, all porous PEEK cubes were carefully polished using a mechanical polishing machine to ensure flat parallel sample surfaces. Samples with obvious cracks or voids were discarded from the measurements. At least 5 samples were compressed to 75% of their original height at a test speed of 1 mm/min, and engineering stress strain curves recorded. The elastic modulus (*E*) was determined from the linear elastic region of the stress–strain curve. The crush strength (*σ*_c_) is defined as the highest strength at the end of the initial linear elastic region (see **SI** Figure S3).

The **glass transition temperature** *T*_g_ and **degree of crystallinity** *X*_*DSC*_ were determined using differential scanning calorimetry (DSC, Discovery series, TA instruments). Between 3 and 6 mg of each sample was analyzed from 50–400°C at a heating rate of 10°C/min. The heat flow was recorded twice for two subsequent heating and cooling runs. *X*_*DSC*_ was determined from the area under the melting peak of the first heating run as follows:

(2) $$X_{\mathrm{DSC}}=\frac{\Delta H_{f}^{\text{peak}}}{\Delta H^{0}}$$

where *Δ* $H_{f}^{\text{peak}}$ is the measured melting enthalpy and *ΔH* ^*0*^ the theoretical enthalpy of melting of a perfectly crystalline PEEK sample (130 J/g).^34^

Selected samples (**A1**, **B1**, **B4**, **C2**, and **D1**) were investigated using ultra‐fast calorimetry using a **Flash‐DSC** (DSC 2+ Mettler Toledo equipped with UFS 1 MEMS sensors). Such experiments allow for heating and cooling rates of up to 40,000 and 10,000 K/s, respectively, which allows the suppression of secondary crystallization during the experiment.

The critical cooling rate to suppress PEEK crystallization is in the order of 2 K/s.^35^ Therefore, by melting and subsequent quenching at rates of 5000 K/s fully amorphous PEEK can be created and the step in the heat flux related to the glass transition can be evaluated. The step of the heat flux associated with the glass transition of the fully amorphous state was used to calibrate the mass of the sample. Heat losses are independent of the heating rate β^36^ and cancel out in the difference of the step height. Hence, these can be used for the determination of the sample mass on the FDSC chip *m*_*FDSC*_:

(3) $$m_{\text{FDSC}}=\frac{\Delta \phi_{\beta }\;}{\beta \;\Delta c_{p}}$$

where *Δφ*_*β*_ is the step of the heat flux at *T*_g_ determined at a heating rate β (= 5000 K/s) and *Δc*_*p*_ (= 0.271 J/g K^−1^) the step of the heat capacity at *T*_g_ of PEEK as reported by Cheng et al.^37^ Sample masses ranged from 5 to 120 ng.

**Surface area** and mesopore size distribution were determined by nitrogen adsorption at 77 K using the Brunauer–Emmet–Teller (BET) and Barrett–Joyner–Halenda (BJH) methods performed in a surface area analyzer (TriStar II 3020, Micromeritics Ltd., Norcross). Samples with total mass of 0.2 to 0.4 g, were cut or broken into small pieces (roughly 1–2 mm in all dimensions) and dried at 100°C under active nitrogen flow for 12 h prior to measurement.

To determine whether 4PPH remained in PEEK, we performed a **qualitative phenol test**.^38^ Seven test tubes with 7 various solutions were prepared. Test tubes were filled with pure acetone or acetone extracted original PEEK as negative controls, 0.5 g of 4PPH were dissolved in acetone as a positive control or extracts from ground PEEK foams (**A1**, **A4**, **D1**, and **D4**). The original or ground PEEK foams were dispersed in acetone and shaken for 24 h. Afterwards, the acetone used to extract the PEEK was collected and added to test tubes and finally 0.3 ml of 1 wt% iron(III) chloride solution was added to each tube and shaken vigorously (see **SI** Figure S4).

## 3 RESULTS AND DISCUSSION

**FIGURE 2:**
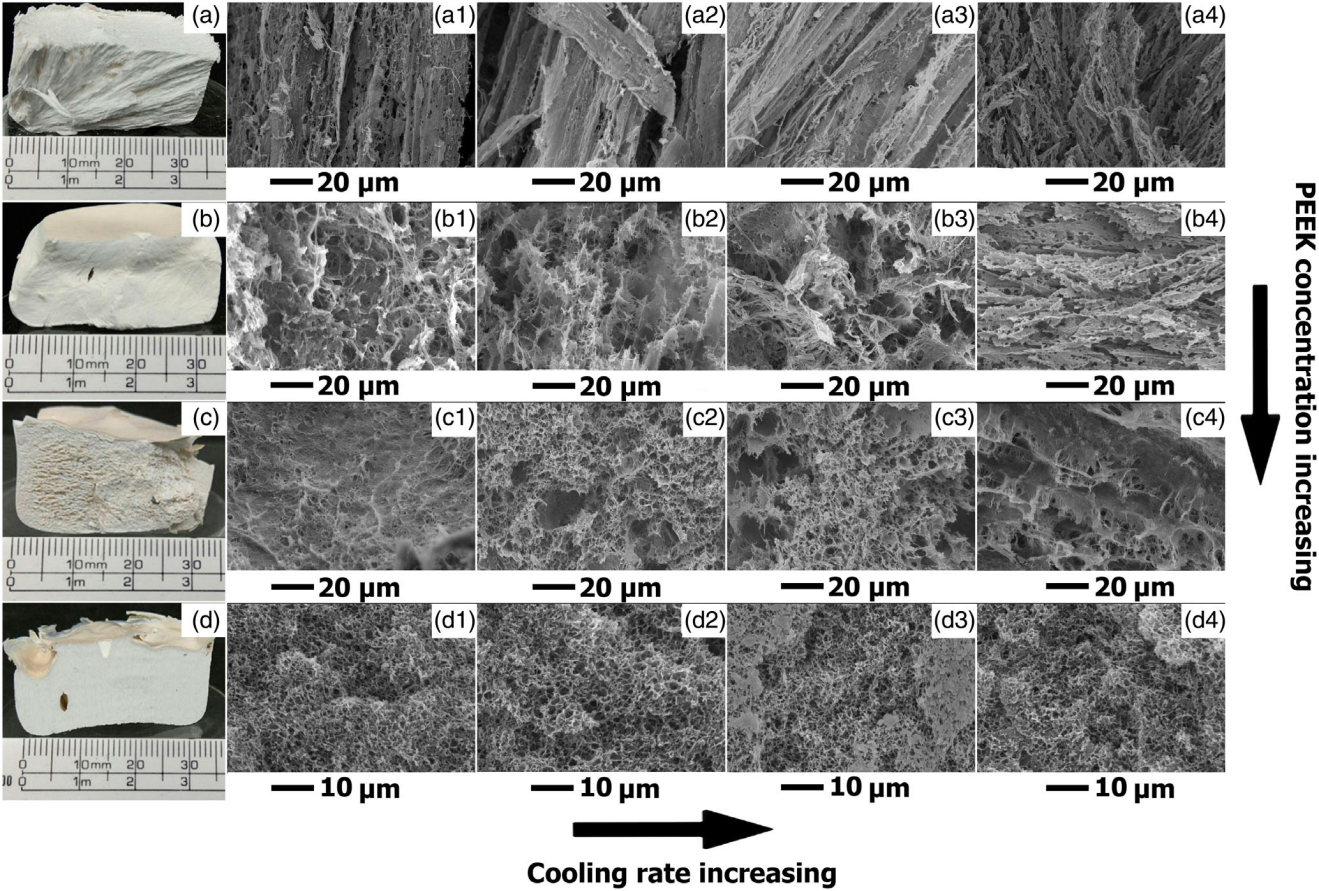
Representative photographs and SE micrographs of PEEK foams prepared from PEEK in 4PPH solutions containing 5 wt% (a), 10 (b), 15 (c), and 20 wt% (d) PEEK at cooling rates β = 0.5°C/min (1), 1°C/min (2), 2°C/min (3), and 10°C/min (4). (×3000 magnification). Please note that for row **D** we used a higher magnification [Color figure can be viewed at wileyonlinelibrary.com]

Table 1 summarizes our observations during the cooling process. During cooling of 5 wt% PEEK in 4PPH solutions using the HT‐TIPS process at all cooling rates, we observed a fast solid–liquid demixing (type 1) in the temperature range of 141–151°C. Typically, solvent crystallization occurred in a few seconds (Table 1), immobilizing the PEEK (Video **S1** **‐A1, SI**) and yielding anisotropic, fiber‐like macro‐ and microscopic structures (photographs and SE micrographs in Figure 2). These samples were feeble and difficult to handle because of weak interactions between the fiber‐like features (Figure 2 row **A**). Solvent crystallization forced the PEEK out of solution and the crystals grew in the freezing direction of the solvent, which formed the anisotropic fiber‐like structure, typical for porous polymers obtained by TIPS processes where solvent crystallization occurred before solution gelation.^42^, ^45^ The foams obtained using a cooling rate of 0.5°C/min (**A1**) contained long, thin fibers with no apparent fiber‐connectivity. The fiber ends have sharp edges and look as if they were torn apart. Foams prepared at cooling rates of 1 (**A2**) and 2°C/min (**A3**) have a similar morphology, but the fibers appear more densely packed. In foams prepared at a cooling rate of 10°C/min (**A4**) the fibers were much shorter and more chaotically oriented. The foams produced from 5 wt% PEEK in 4PPH solutions had channel‐like pores with the largest diameter.

In contrast, slow solid–liquid demixing prior to solvent crystallization (type 2) was observed during cooling of 20 wt% PEEK solutions (Video S2 **‐D1, SI**). During this demixing process the PEEK solutions became increasingly turbid and gelled. Gelation is caused by (1) decreasing polymer solubility in solutions with decreasing temperature and (2) polymer crystallization from solution becoming more preferable for semicrystalline polymers as the solution temperature drops below their crystallization temperature.^39^ The gelation temperature decreased with increasing cooling rates (Table 1). Lower cooling rates provided enough time for PEEK molecules to crystallize from solution thus creating a gel‐like structure. The influence of cooling rate on PEEK crystallization process from melt has been well described in the literature^40^, ^41^ and thus it is fair to assume that the same behavior exists for crystallization of PEEK from solution. The gel structure was frozen upon reaching the solvent crystallization temperature of 4PPH. All porous PEEK obtained by HT‐TIPS from 20 wt% PEEK in 4PPH solutions had a smooth, white surface (Photo Figure 2 row **D**) and, at the microscale, a uniform morphology at all cooling rates (Figure 2, **D1‐D4**), producing the smallest pores of all our PEEK foams. The high gelation point (see Table 1) and long time between gelation and solvent crystallization resulted in a homogeneous pore structure (Figure 2, **D1‐D4**).

At concentrations between the two extremes, one observes an overlap of type 1 and 2 solid–liquid demixing processes. As shown in Figure 2 row **B** and **C**, the PEEK foams obtained from solutions containing 10 and 15 wt% PEEK had, when produced at low cooling rates (β = 0.5 and 1°C/min), a typical homogeneous pore structure (Figure 2 **B1** and **C1**, **2**). At increased cooling rates (β = 2 and 10°C/min) a mixed fiber‐like/pore structure was obtained (Figure 2 **B2‐4** and **C3**, **4**). It is worth noting that at β = 10°C/min, gelation and solution crystallization occurred too fast to visibly distinguish between the two processes (Video S3 **‐A4, SI**).

As demonstrated above, the polymer concentration greatly affected the pore structure. Generally, with increasing polymer concentration the pore size decreased, and the foam morphology became more homogeneous (Figure 2).

For all PEEK foams type IV BET isotherms (**SI** **Figure S5**) were obtained. Type IV isotherms are indicative of meso/macroporous materials containing pores with diameters ranging from 2 to 100 nm. All foams displayed well‐pronounced hysteresis loops and thus it is fair to assume that cylindrical pores were more prominent in the pore walls of the samples than ink‐bottle shaped pores. The specific surface areas of porous PEEK produced were in the range of 132–173 m^2^/g (see Table 2). Three phases form when PEEK crystallization starts from a homogeneous solution: a crystalline PEEK phase, an amorphous PEEK phase still containing solvent, and a solvent‐rich phase containing some dissolved polymer. Thus, it is likely that the mesopores in the pore walls of the PEEK foams formed during the extraction of the crystalline 4PPH by ethanol and are the remnants of the polymer remaining in solvent‐rich phase. The porosity of the PEEK foams (Table 2) did not significantly affect the surface area. Interestingly, the cooling rate did appear to affect the surface area; lower cooling rates yielded higher specific surface areas within the PEEK foams. This effect could potentially be due to pores collapsing during rapid cooling or solvent crystallization.

**TABLE 2:** Skeletal $\rho_{s}$ and foam density $\;\rho_{f}$, porosity P and BET surface area A_s_ of PEEK foams

	[PEEK]/wt%				
β/°C/min	Property	5	10	15	20
**0.5**	$\rho_{s}$/[g/cm^3^]	1.38 ± 0.03	1.38 ± 0.02	1.38 ± 0.02	1.37 ± 0.01
	$\rho_{f}$/[g/cm^3^]	0.11 ± 0.02	0.2 ± 0.04	0.27 ± 0.07	0.36 ± 0.06
	P/%	92 ± 1	86 ± 2	81 ± 5	74 ± 4
	*A*_*s*_/m^2^/g	159 ± 7	160 ± 10	170 ± 30	140 ± 10
**1**	$\rho_{s}$ [g/cm^3^]	1.37 ± 0.03	1.38 ± 0.02	1.37 ± 0.01	1.37 ± 0.02
	$\rho_{f}$ [g/cm^3^]	0.16 ± 0.06	0.17 ± 0.02	0.29 ± 0.08	0.36 ± 0.04
	P [%]	89 ± 4	88 ± 1	79 ± 6	73 ± 4
	*A*_*s*_/m^2^/g	151 ± 2	150 ± 10	150 ± 20	140 ± 20
**2**	$\rho_{s}$ [g/cm^3^]	1.37 ± 0.04	1.37 ± 0.01	1.37 ± 0.01	1.38 ± 0.01
	$\rho_{f}$ [g/cm^3^]	0.12 ± 0.06	0.15 ± 0.02	0.24 ± 0.02	0.32 ± 0.06
	P [%]	91 ± 4	89 ± 3	83 ± 2	75 ± 7
	*A*_*s*_/m^2^/g	140 ± 4	154 ± 2	149 ± 3	145 ± 4
**10**	$\rho_{s}$ [g/cm^3^]	1.36 ± 0.01	1.37 ± 0.01	1.38 ± 0.01	1.37 ± 0.01
	$\rho_{f}$ [g/cm^3^]	0.14 ± 0.04	0.13 ± 0.03	0.21 ± 0.08	0.29 ± 0.06
	P [%]	90 ± 3	91 ± 2	85 ± 5	79 ± 4
	*A*_*s*_/m^2^/g	130 ± 20	137 ± 1	149 ± 4	141 ± 5

**FIGURE 3:**
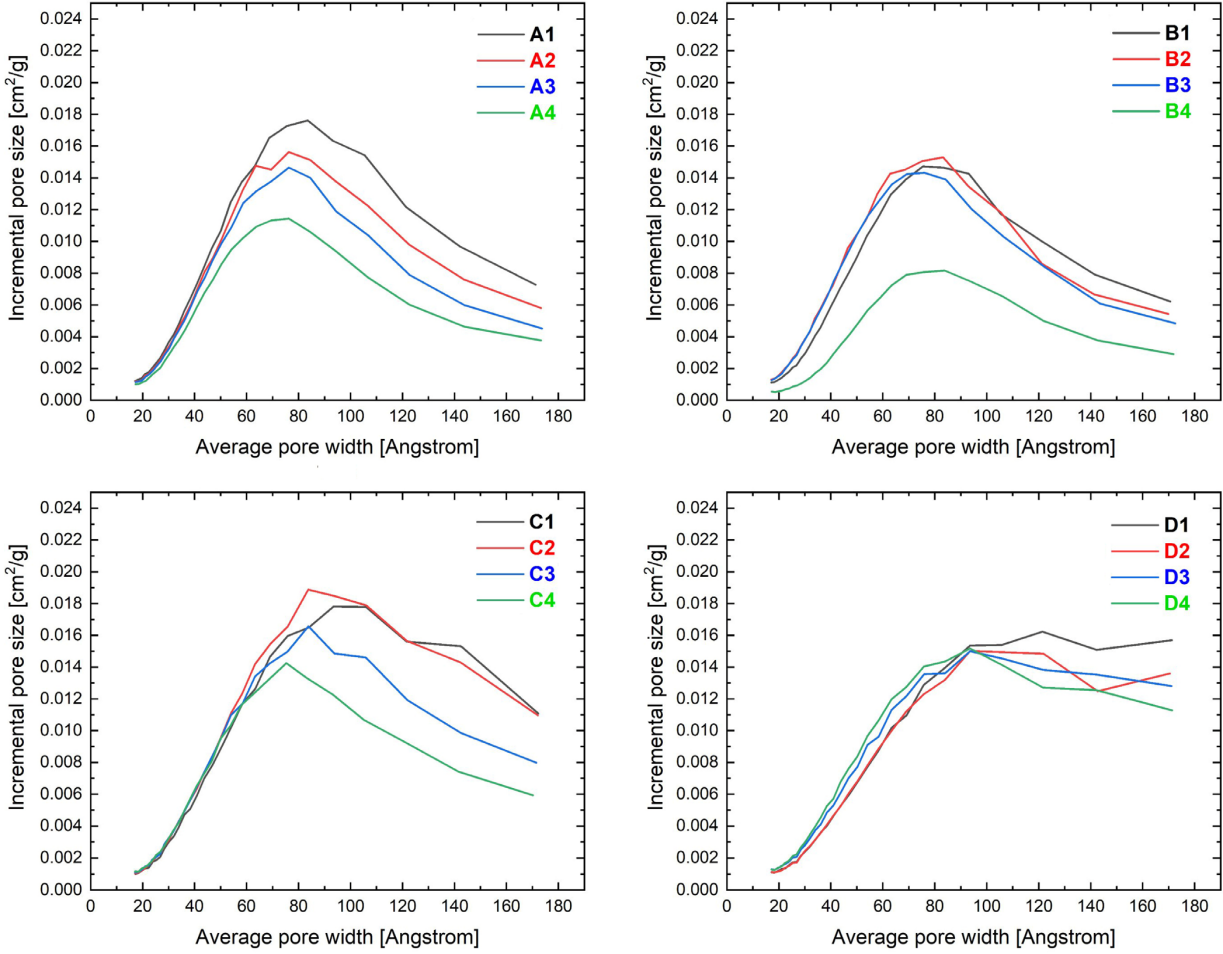
Incremental pore size distribution as function of average pore width for 5 wt% (A1‐A4), 10 wt% (B1‐B4), 15 wt% (C1‐C4), and 20 wt% (D1‐D4) foamed PEEK [Color figure can be viewed at wileyonlinelibrary.com]

The total specific surface area is the sum of the surface areas resulting from large macropores in the foams and mesoscale porosity present in the pore walls of the PEEK foams. The Barrett–Joyner–Halenda (BJH) method was used to determine the pore‐size distribution resulting from mesopore scale pores. Besides the macropores visible in the SEM (Figure 2) the foams also contained mesopores with sizes ranging from 2 to 18 nm. Figure 3 shows the pore size distributions for the produced porous PEEK with varying porosities.

The foam density and thus porosity of the PEEK foams is controlled by the PEEK concentration in the solution; increasing the PEEK concentration results in an increased foam density and lower porosity (Table 2), as may be expected. The cooling rate did not significantly affect the foam and skeletal density. However, porous PEEK produced from 5 and 10 wt% solutions obtained at a cooling rate of 10°C/min had a higher porosity than expected. A possible explanation for this could be the high sample anisotropy of all specimens obtained at high cooling rates (Figure 2).

The measured skeletal densities of porous PEEK produced from solutions containing increasing PEEK concentrations using different cooling rates ranged from 1.36 to 1.38 g/cm^3^ (Table 2). The amorphous and crystalline phase of PEEK have different densities and thus the absolute density of PEEK depends on the amorphous/crystalline phase ratio. Fully amorphous PEEK has a density of around 1.26 g/cm^3^,^43^ while fully crystalline PEEK has a density close to 1.40 g/cm^3^.^44^ Therefore, our results suggest that the volume fraction degree of crystallinity of the PEEK in our foams ranges from 71% to 86%. This is somewhat surprising as PEEK, when melt processed, has typically a degree of crystallinity of ~30%–40%, which is primarily influenced by processing conditions and thermal history.^40^

**FIGURE 4:**
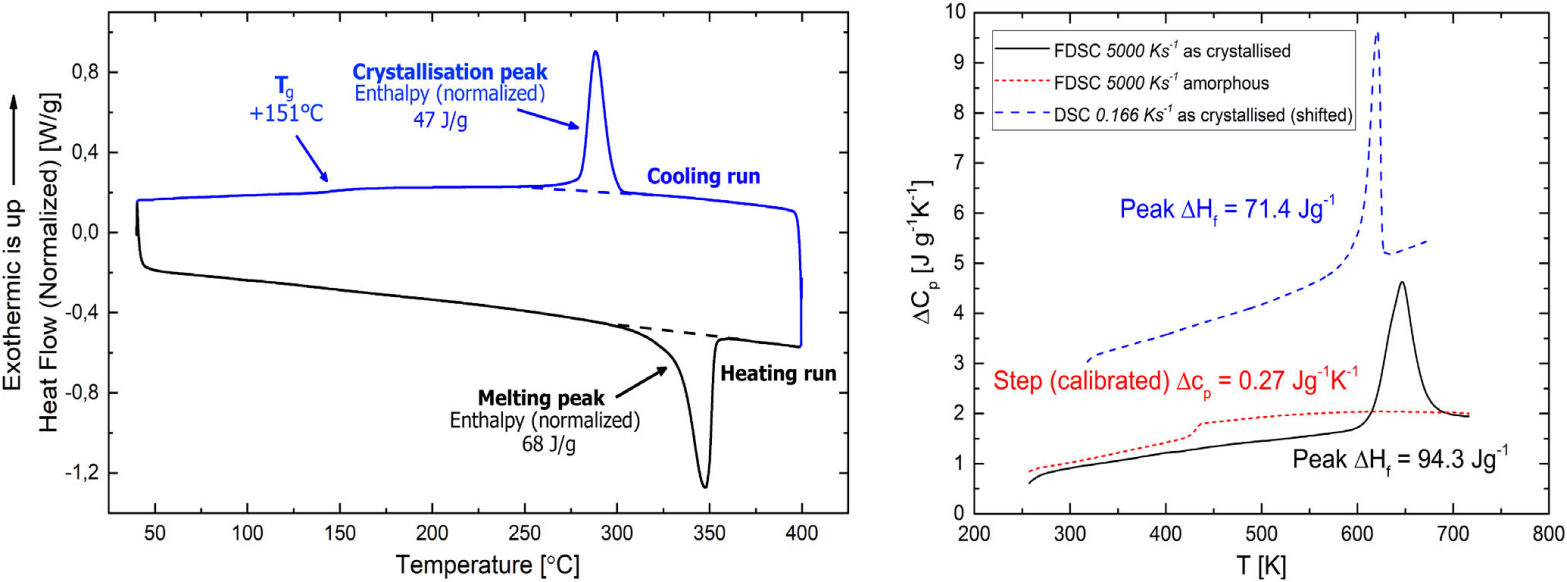
DSC (left figure) and flash DSC (right figure) evaluation of crystallinity sample **A1** [Color figure can be viewed at wileyonlinelibrary.com]

However, in our study PEEK (powder) was fully dissolved at ~250–270°C in the high boiling solvent 4PPH and then crystallized from solution during the cooling process. The thermal properties were characterized by DSC, shown in Figure 4.

The glass transition temperature of (APC‐2) PEEK, according to the manufacturer, ought to be +143°C with minor variations and should be evident as a step in the DSC heating curve.^15^ However, for semicrystalline polymers with a high degree of crystallinity, *T*_g_ can be difficult to define. Our porous PEEK, which crystallized from 4PPH solution during cooling (see Table 1), possessed rather high degrees of crystallinities, higher than those reported in the literature,^40^, ^41^, ^50^ with values exceeding 50% and, therefore, no well‐visible glass transition step was discernible in the heating run (Figure 4(a)). The degree of crystallinities determined from the recrystallisation peak (now from the melt) during the cooling run, were below 40%, as expected. In addition, the glass transition step during cooling was visible (Figure 4(a)). To ensure that these high degrees of crystallinity determined by standard DSC experiments were correct they were remeasured by Flash‐DSC (Figure 4(b)). Degrees of crystallinity, $X_{\mathrm{DSC}}$, for our porous PEEKs are listed in **Table** **3**.

**TABLE 3:** Measured change in melt and crystallization enthalpies ΔH_f_ and degrees of crystallinity $X_{\mathrm{DSC}}$, calculated using equation 3, obtained by standard DSC of porous PEEK

	ΔH_f_ [J/g]		$X_{\mathrm{DSC}}$ [%]	
Sample	Heating run	Cooling run	Heating run	Cooling run
**A1**	70 ± 2	47 ± 2	54 ± 2	40 ± 1
**A2**	70 ± 2	48 ± 1	54 ± 2	40 ± 1
**A3**	66 ± 2	49 ± 1	50 ± 2	38 ± 1
**A4**	64 ± 1	49 ± 2	49 ± 1	38 ± 2
**B1**	71 ± 2	49 ± 2	54 ± 2	38 ± 1
**B2**	71 ± 2	52 ± 1	55 ± 1	40 ± 1
**B3**	69 ± 4	53 ± 1	53 ± 3	41 ± 1
**B4**	63 ± 4	50 ± 3	48 ± 3	38 ± 3
**C1**	72 ± 1	47 ± 1	55 ± 1	36 ± 1
**C2**	71 ± 1	53 ± 1	55 ± 1	41 ± 1
**C3**	71 ± 1	53 ± 1	55 ± 1	41 ± 1
**C4**	66 ± 2	53 ± 2	51 ± 2	41 ± 2
**D1**	73 ± 3	52 ± 1	56 ± 2	41 ± 1
**D2**	74 ± 1	54 ± 1	57 ± 1	42 ± 1
**D3**	71 ± 2	52 ± 1	55 ± 1	40 ± 1
**D4**	68 ± 5	51 ± 1	52 ± 4	39 ± 1

No substantial recrystallisation is possible when heating with rates of 5000 K/s as the heating curve of the fully amorphous state clearly shows (no endothermic peak in the red curve in Figure 4(b)). Also, no melting or recrystallisation is discernible in the as‐crystallized state (black curve in Figure 4(b)). The degree of crystallinity of the five selected samples was determined from the heat of fusion of as‐crystallized samples with good precision by division with the heat of fusion of the fully crystalline state (ΔH_f_ = 130 J/g). The crystallinity determined by FDSC confirms the high crystallinities obtained by standard DSC measurements (Table 3). However, FDSC also shows that the crystallinity was underestimated by 10%–15% during standard DSC measurements (Table 4). FDSC allows for the inhibiting of dynamic melting‐recrystallization during heating, which affects the reliability of the interpretation of the melting peak in standard DSC. Flash‐DSC, therefore, better reflects the crystallinity of the as‐produced state, which is in much better agreement with the volumetric crystallinities estimated from measured densities. The high degree of crystallinity $X_{\text{FDSC}}$ did positively influence the mechanical strength of the produced PEEK foams.

**TABLE 4:** Degree of crystallinity $X_{\text{FDSC}}$ calculated from ΔH_f_ data, determined by FDSC measurements

Sample	$X_{\text{FDSC}}$ [%]	Sample mass [ng](calibrated through *Δφβ*)
**A1**	72	38.4
**B1**	64	5.3
**B4**	61	46.1
**C2** (small load)	59	8.5
**C2** (heavy load)	61	118.1
**D1**	76	40.4

The PEEK amorphous phase tends to transform to a crystalline phase at temperatures above *T*_g_, a well‐known effect called polymer annealing. Annealing of PEEK is well documented in the literature and occurs at temperatures between *T*_g_ and the melting point.^47^, ^48^ In this temperature range, polymer chains become more mobile and tend to assume a more compact state to reduce the system energy, joining the crystal lattice at temperatures above *T*_g_. PEEK annealing generally starts at temperatures above 200°C with annealing time being an important parameter due to the slow polymer chain rearrangement process.^49^ In general, longer annealing times result in a higher degree of crystallinity.

Based on these facts and our findings, we hypothesize the following explanation of the observed effects: (i) dissolving PEEK in 4PPH at temperatures above *T*_g_ is increasing the mobility of polymer chains during cooling, which is the main factor for the high degree of crystallinity (Table 4), (ii) cooling PEEK in 4PPH solution during the HT‐TIPS process, leads to PEEK crystallization from solution; lower cooling rates allow for longer crystallization times at temperatures above *T*_g_, which results in a higher degree of crystallinity.

The amorphous/crystalline phase ratio of a polymer affects density, thermal behavior, solvent resistance, processability, and mechanical properties. Higher degrees of crystallinity result in close packing of the PEEK molecules, which results in a higher skeletal density and correspondingly higher elastic moduli, as our results confirm.

**FIGURE 5:**
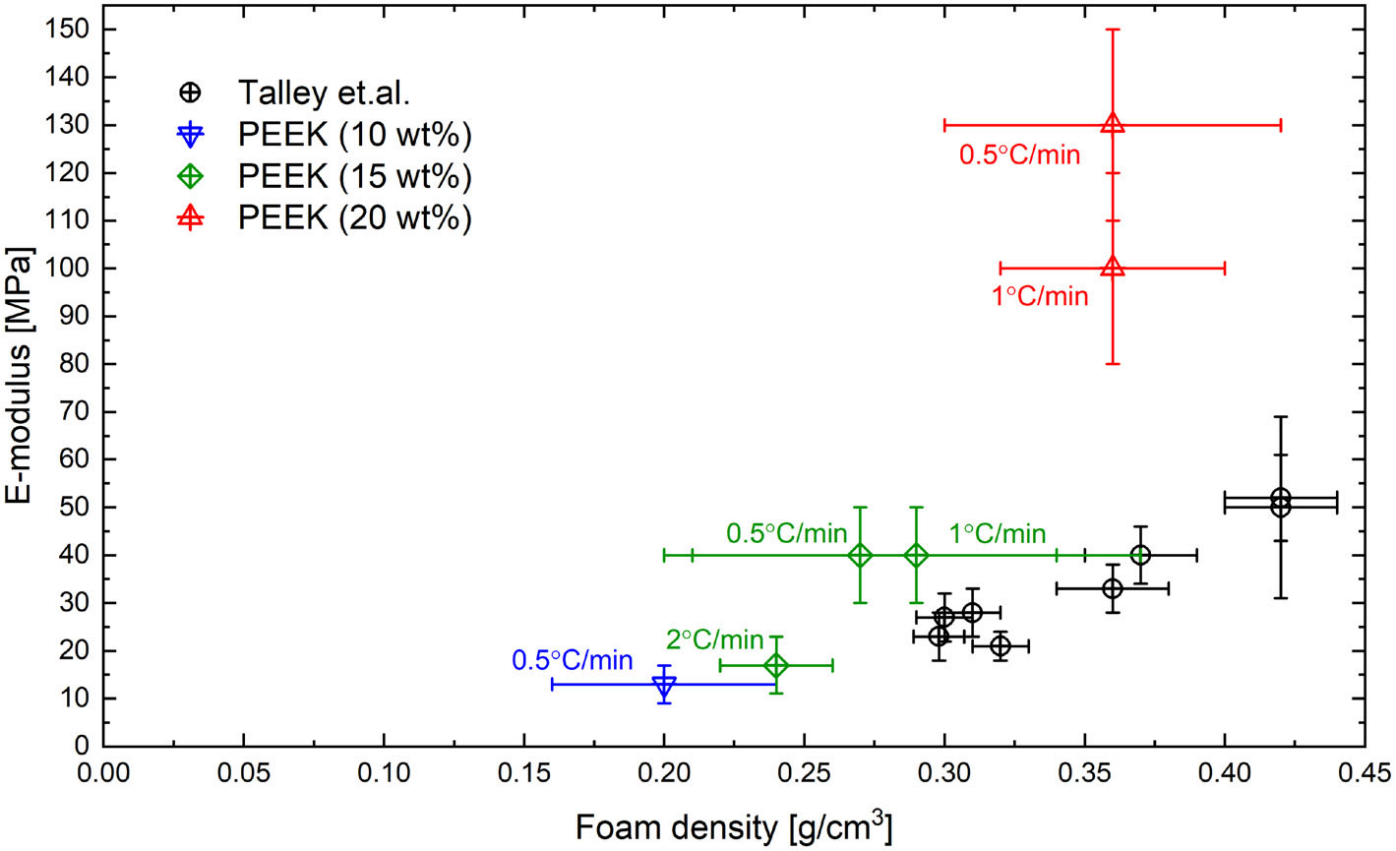
Elastic moduli of our porous PEEK obtained using various cooling rates and polymer concentrations compared with literature data.^31^ Detailed graphs showing E‐moduli and crush strengths as a function of foam density and porosity of our PEEK samples can be found in figure S7 (SI) [Color figure can be viewed at wileyonlinelibrary.com]

The mechanical properties of the foams prepared from 5 wt% PEEK in 4PPH solutions could not be determined due to their feeble, anisotropic, fiber‐like structure, which made it impossible to prepare test specimens. Of the foams prepared from 10 wt% PEEK in 4PPH solutions, only the sample produced using a cooling rate of 0.5°C/min could be cut and tested. This sample had a crush strength of 0.8 MPa and an elastic modulus of 13 MPa (Table 5) despite its low foam density and high porosity (88%), which was caused by its relatively homogeneous morphology (Figure 2, **B1**). The foams produced from 15 wt% PEEK in 4PPH solutions had similar porosities (ranging between 79% and 85%) but much higher E‐moduli and crush strengths compared to the foams prepared from 10 wt% solutions. The mechanical properties of the samples produced using cooling rates of 0.5 (**C1**) and 1°C/min (**C2**) were considerably higher than those of foams produced with a cooling rate of 2°C/min (**C3**) (Figure 5). This behavior can be explained by the change of the foam morphology; the PEEK foam **C1** and **C2** were obtained by type 2 solid/liquid demixing and had, therefore, homogeneous pore structures while for **C3** the shorter gelation time caused it to demix in a type 1/2 mixed‐mode resulting in a mixed fiber‐like/pore structure. The foams (**C4**) produced using 15 wt% PEEK in 4PPH using a cooling rate of 10°C/min contained large cracks (see Figure S6, **С4**), which did not allow for the preparation of test specimens.

**TABLE 5:** Mechanical properties of the foamed PEEK samples obtained from various PEEK concentrations (5 wt% samples are not possible to cut for compression tests) and cooling rates (10°C/min samples are not available because of thermo‐residual stress cracks presents), where E represents elastic modulus and σ is crush strength

	[PEEK]/wt%			
β/°C/min	Property	10	15	20
**0.5**	E [MPa]	13 ± 4	40 ± 10	130 ± 20
	σ [MPa]	0.8 ± 0.1	1.8 ± 0.6	6.6 ± 0.8
**1**	E [MPa]	n/a	40 ± 10	100 ± 20
	σ [MPa]	n/a	1.9 ± 0.1	7 ± 1
**2**	E [MPa]	n/a	17 ± 6	n/a
	σ [MPa]	n/a	1.9 ± 0.1	n/a

The foams prepared from 20 wt% PEEK solutions possessed the highest mechanical properties of all porous PEEK samples; those foams made with cooling rates of 0.5 (**D1**) and 1°C/min (**D2**) had elastic moduli up to 125 MPa and crush strength of 6–7 MPa. Note that the foam density, porosity (Table 2) and morphology of **D1** and **D2** (Figure 2, **D1‐2**) were rather similar and, therefore, also their mechanical properties. However, compared to the B and C‐series the foam density increased significantly, and even more importantly, the degree of crystallinity increased by about 12% (Table 4), which led to the much‐improved mechanical performance. Unfortunately, it was impossible to produce test specimens from foams produced at higher cooling rates (2 and 10°C/min) as they contained many internal cracks, likely caused by residual thermal stresses^46^ (Figure S6, D3‐4).

Figure 5 shows also the elastic moduli of PEEK foams reported by Talley et al.^31^ The porous PEEK produced by us had at much lower foam densities, yet similar elastic moduli compared to those reported by Talley et al. This is possibly due to the higher degree of crystallinity of PEEK crystallized from 4PPH solution as compared to PEEK processed from dichloroacetic acid (maximum crystallinity = 51%).^31^

## 4 CONCLUSIONS

High porosity PEEK foams with controllable porosity were produced using an HT‐TIPS process. The influence of polymer concentration and cooling rate was investigated. Based on the findings, two main conclusions can be drawn: (1) polymer concentration significantly affects the demixing process – at PEEK concentrations in 4PPH below 10 wt%, solvent solidification occurs prior to polymer demixing while at higher polymer concentrations, solution gelation caused by PEEK crystallization takes place before solvent crystallization. The nature of the liquid–solid phase separation process determines the pore structure and morphology. The foam density (110 to 360 kg/m^3^) and thus porosity (92% to 73%) of our PEEK foams can be controlled by the amount (up to 20 wt%) of PEEK dissolved in 4PPH. The crystallization time of PEEK from 4PPH solutions is controlled by the cooling rate and results in very high crystallinity PEEK in our foams, which both lead to much higher skeletal PEEK densities (1360–1380 kg/m^3^) and elastic moduli of up to 125 MPa (as well as crush strengths).

## Supporting information


Appendix S1: Supporting Information

**Video S1** 

**Video S2** 

**Video S3** 

## References

[1] J. Da Silva Burgal , L. G. Peeva , S. Kumbharkar , A. Livingston , J. Membr. Sci. 2015, 479, 105.

[2] G. Wang, High Performance Polymers and Engineering Plastics (Eds: Vikas Mittal), John Wiley & sons, Hoboken, New Jersey 2011. https://onlinelibrary.wiley.com/doi/10.1002/9781118171950.ch10.

[3] V. Mylläri , T.‐P. Ruoko , J. Vuorinen , H. Lemmetyinen , Polym. Degrad. Stab. 2015, 120, 419.

[4] D. Li , D. Shi , K. Feng , X. Li , H. Zhang , J. Membr. Sci. 2017, 530, 125.

[5] H. N. Beck , J. Appl. Polym. Sci. 1992, 45, 1361.

[6] M. F. Sonnenschein , J. Polym. Sci., Part B: Polym. Phys. 2003, 41, 1168.

[7] P. Venkatraman , C. Rader , N. Bohmann , E. J. Foster , Polymer 2019, 169, 154.

[8] F. Li , Y. Hu , X. Hou , X. Hu , D. Jiang , High Perform. Polym. 2017, 30, 657.

[9] C. Y. Hsu , K. Scrafford , C. Ni , F. Deng , Polym. Eng. Sci. 2019, 59, 1209.

[10] L. Q. Cortes , A. Lonjon , E. Dantras , C. Lacabanne , J. Non‐Cryst. Solids 2014, 391, 106.

[11] J. Cao , Y. Lu , H. Chen , L. Zhang , C. Xiong , Int. J. Polym. Mater. Polym. Biomater. 2018, 68, 433.

[12] S. Najeeb , M. S. Zafar , Z. Khurshid , F. Siddiqui , J. Prosthodont. Res. 2016, 60, 12. 26520679 10.1016/j.jpor.2015.10.001

[13] I. V. Panayotov , V. Orti , F. Cuisinier , J. Yachouh , J. Mater. Sci. Mater. Med. 2016, 27, 118. 27259708 10.1007/s10856-016-5731-4

[14] M. F. Sonnenschein , J. Appl. Polym. Sci. 1999, 72, 175.

[15] D. Parker, J. Bussink, H.T. Van De Grampel, G.W. Wheatley, E.‐U. Dorf, E. Ostlinning, K. Reinking, F. Schubert, O. Jünger, Ullmann's Encyclopedia of Industrial Chemistry. John Wiley & Sons, Hoboken, New Jersey 2012. https://onlinelibrary.wiley.com/doi/10.1002/14356007.a21_449.pub4.

[16] C. Barile , C. Casavola , F. De Cillis , Composites, Part B 2019, 162, 122.

[17] А. А. Stepashkin , D. I. Chukov , F. S. Senatov , A. I. Salimon , A. M. Korsunsky , S. D. Kaloshkin , Compos. Sci. Technol. 2018, 164, 319.

[18] Q. Liu , Z. Tang , B. Ou , L. Liu , Z. Zhou , S. Shen , Y. Duan , Mater. Chem. Phys. 2014, 144, 213.

[19] I. Pulko , V. Smrekar , A. Podgornik , P. Krajnc , J. Chromatogr. A 2011, 1218, 2396. 21168141 10.1016/j.chroma.2010.11.069

[20] M. Tebboth , A. Menner , A. Kogelbauer , A. Bismarck , Curr. Opin. Chem. Eng. 2014, 4, 114.

[21] N. T. Evans , F. B. Torstrick , C. S. Lee , K. M. Dupont , D. L. Safranski , W. A. Chang , A. E. Macedo , A. S. Lin , J. M. Boothby , D. C. Whittingslow , R. A. Carson , R. E. Guldberg , K. Gall , Acta Biomater. 2015, 13, 159. 25463499 10.1016/j.actbio.2014.11.030 PMC4294703

[22] P. Werner , R. Verdejo , F. Wöllecke , V. Altstädt , J. K. W. Sandler , M. S. P. Shaffer , Adv. Mater. 2005, 17, 2864.

[23] A. R. Siddiq , A. R. Kennedy , Mater. Sci. Eng. C Mater. Biol. Appl. 2015, 47, 180. 25492187 10.1016/j.msec.2014.11.044

[24] R. Verdejo , P. Werner , J. Sandler , V. Altstädt , M. S. P. Shaffer , J. Mater. Sci. 2009, 44, 1427.

[25] L. Cai , Y. Pan , S. Tang , Q. Li , T. Tang , K. Zheng , A. R. Boccaccini , S. Wei , J. Wei , J. Su , J. Mater. Chem. B 2017, 5, 8337. 32264503 10.1039/c7tb02344h

[26] K. H. Tan , C. K. Chua , K. F. Leong , M. W. Naing , C. M. Cheah , Proc. Inst. Mech. Eng. H 2005, 219, 183. 15934394 10.1243/095441105X9345

[27] Q. Yang , G. Zhang , Z. Ma , J. Li , X. Fan , J. Appl. Polym. Sci. 2015, 132, 42576.

[28] L. Cafiero , S. Iannace , L. Sorrentino , Eur. Polym. J. 2016, 78, 116.

[29] I. Akartuna , E. Tervoort , J. C. H. Wong , A. R. Studart , L. J. Gauckler , Polymer 2009, 50, 3645.

[30] S. J. Talley , X. Yuan , R. B. Moore , ACS Macro Lett. 2017, 6, 262. 35650924 10.1021/acsmacrolett.7b00109

[31] S. J. Talley , C. L. AndersonSchoepe , C. J. Berger , K. A. Leary , S. A. Snyder , R. B. Moore , Polymer 2017, 126, 437.

[32] S. J. Talley , S. L. Vivod , B. A. Nguyen , M. A. B. Meador , A. Radulescu , R. B. Moore , ACS Appl. Mater. Interfaces 2019, 11, 31508. 31379150 10.1021/acsami.9b09699

[33] D. Rusakov , A. Menner , A. Bismarck , Macromol. Rapid Commun. 2020, 41, 2000110. 10.1002/marc.202000110 32363705

[34] D. J. Blundell , B. N. Osborn , Polymer 1983, 24, 953.

[35] Y. Furushima , A. Toda , V. Rousseaux , C. Bailly , E. Zhuravlev , C. Schick , Polymer 2016, 99, 97.

[36] C. R. Quick , J. E. K. Schawe , P. J. Uggowitzer , S. Pogatscher , Thermochim. Acta 2019, 677, 12.

[37] S. Z. D. Cheng , M. Y. Cao , B. Wunderlich , Macromolecules 1986, 19, 1868.

[38] W. B. Deichmann , Ind. Eng. Chem. Anal. Ed. 1944, 16, 37.

[39] D. R. Lloyd , K. E. Kinzer , H. S. Tseng , J. Membr. Sci. 1990, 52, 239.

[40] L. Jin , J. Ball , T. Bremner , H.‐J. Sue , Polymer 2014, 55, 5255.

[41] Vasconcelos, G.d.C., R.L. Mazur , E.C. Botelho , M.C. Rezende , and M.L. Costa , J. Aerospace Technol. Manage. 2010, 2, 155.

[42] F. Yang , X. Qu , W. Cui , J. Bei , F. Yu , S. Lu , S. Wang , Biomaterials 2006, 27, 4923. 16759695 10.1016/j.biomaterials.2006.05.028

[43] D. P. Jones , D. C. Leach , D. R. Moore , Polymer 1985, 26, 1385.

[44] C. N. Velisaris , J. C. Seferis , Polym. Eng. Sci. 1986, 26, 1574.

[45] Ö. C. Önder , E. Yilgör , I. Yilgör , Polymer 2016, 107, 240.

[46] W. J. Unger , J. S. Hansen , J. Compos. Mater. 1993, 27, 108.

[47] D. Veazey , T. Hsu , E. D. Gomez , J. Appl. Polym. Sci. 2019, 136, 47727.

[48] Z. Jiang , P. Liu , H.‐J. Sue , T. Bremner , Polymer 2019, 160, 231.

[49] J. Audoit , L. Rivière , J. Dandurand , A. Lonjon , E. Dantras , C. Lacabanne , J. Therm. Anal. Calorim. 2018, 135, 2147.

[50] S.‐L. Gao , J.‐K. Kim , Compos. Part A: Appl. Sci. Manuf. 2000, 31, 517.

