# Supplementary material for: Morphology and properties of foamed high crystallinity PEEK prepared by high temperature thermally induced phase separation
Source: J Appl Polym Sci. 2021 Jul 26;139(1):51423. doi: 10.1002/app.51423 (PMC9286599; doi:10.1002/app.51423)
Supplement: Supplementary file 1 — Appendix S1: Supporting Information [file APP-139-0-s002.docx]

**Morphology and properties of foamed high crystallinity PEEK prepared by high temperature thermally induced phase separation**

Dmitrii Rusakov^a^, Angelika Menner^a^, Florian Spieckermann^b^, Harald Wilhelm^c^ and Alexander Bismarck^a,d,*^

^a^ Institute of Material Chemistry and Research, Polymer and Composite Engineering (PaCE) Group, Faculty of Chemistry, University of Vienna, Währinger Straße 42, 1090 Vienna, Austria

^b^ Materials Physics, Department Materials Science, University of Leoben, 8700 Leoben, Jahnstrasse 12/I

^c^ Laboratory of Polymer Engineering (LKT-TGM), Wexstraße 19-23, 1200 Vienna, Austria

^d^ Department of Chemical Engineering, Imperial College London, South Kensington Campus, London, SW7 2AZ, UK

* corresponding author: alexander.bismarck@univie.ac.at

**Supporting Information
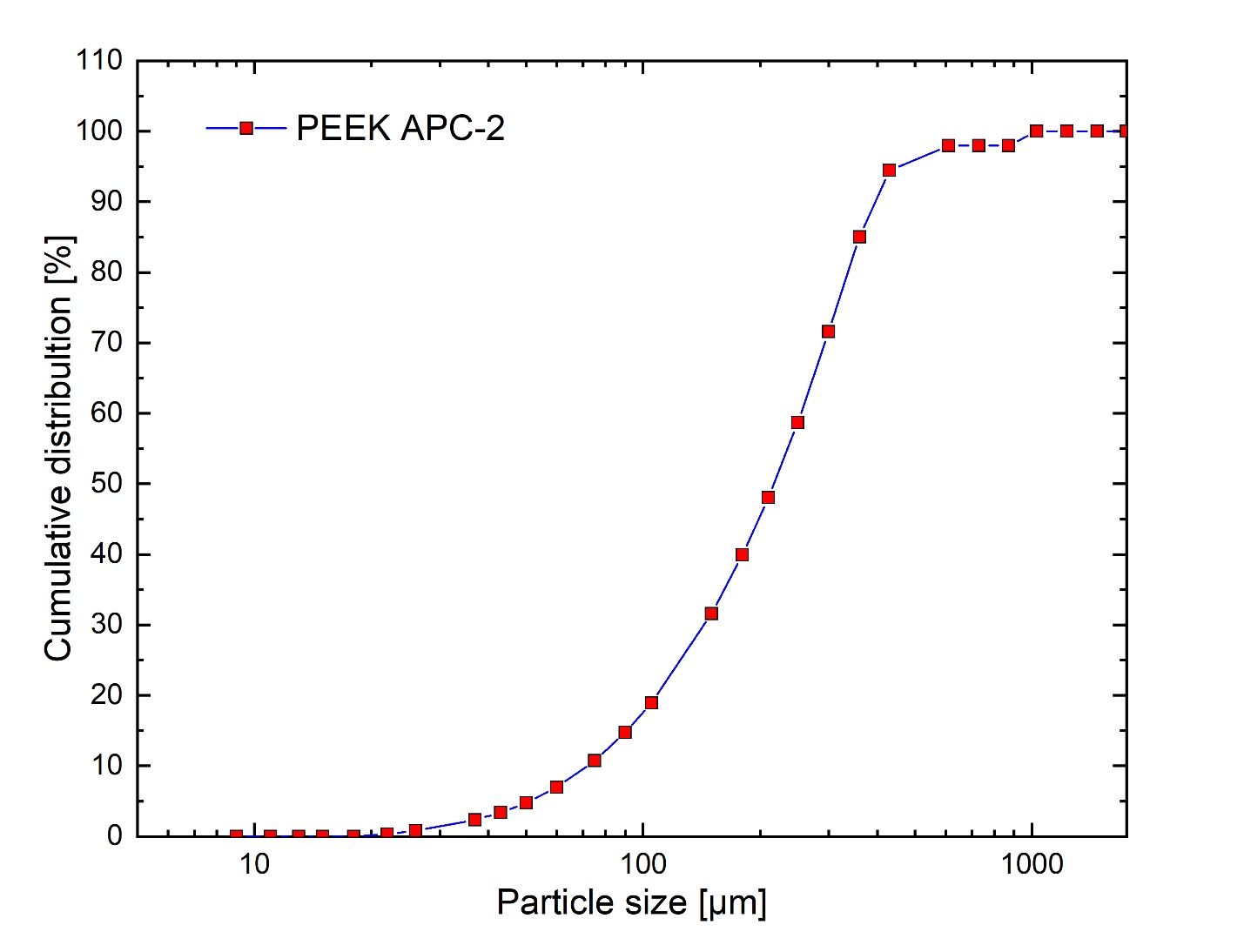
**

**Fig. S1.** *PEEK APC-2 powder cumulative particles size distribution.*

*
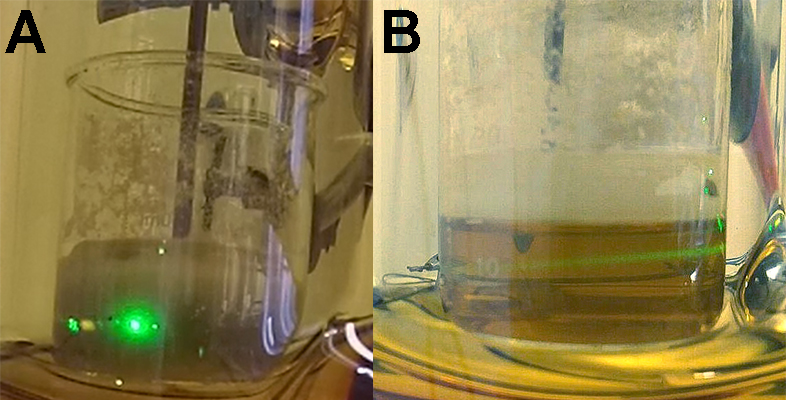
*

**Fig. S2.** *Image (A) represent non-transparent PEEK/4PPH solution with scattered laser pointer spot (****Fig.1*** *point* ***B****, +254°C/77 min of the process); and image (B) with transparent solution, where laser pointer beam going through it without scattering (****Fig.1*** *point* ***C****, +265°C/125 min).*


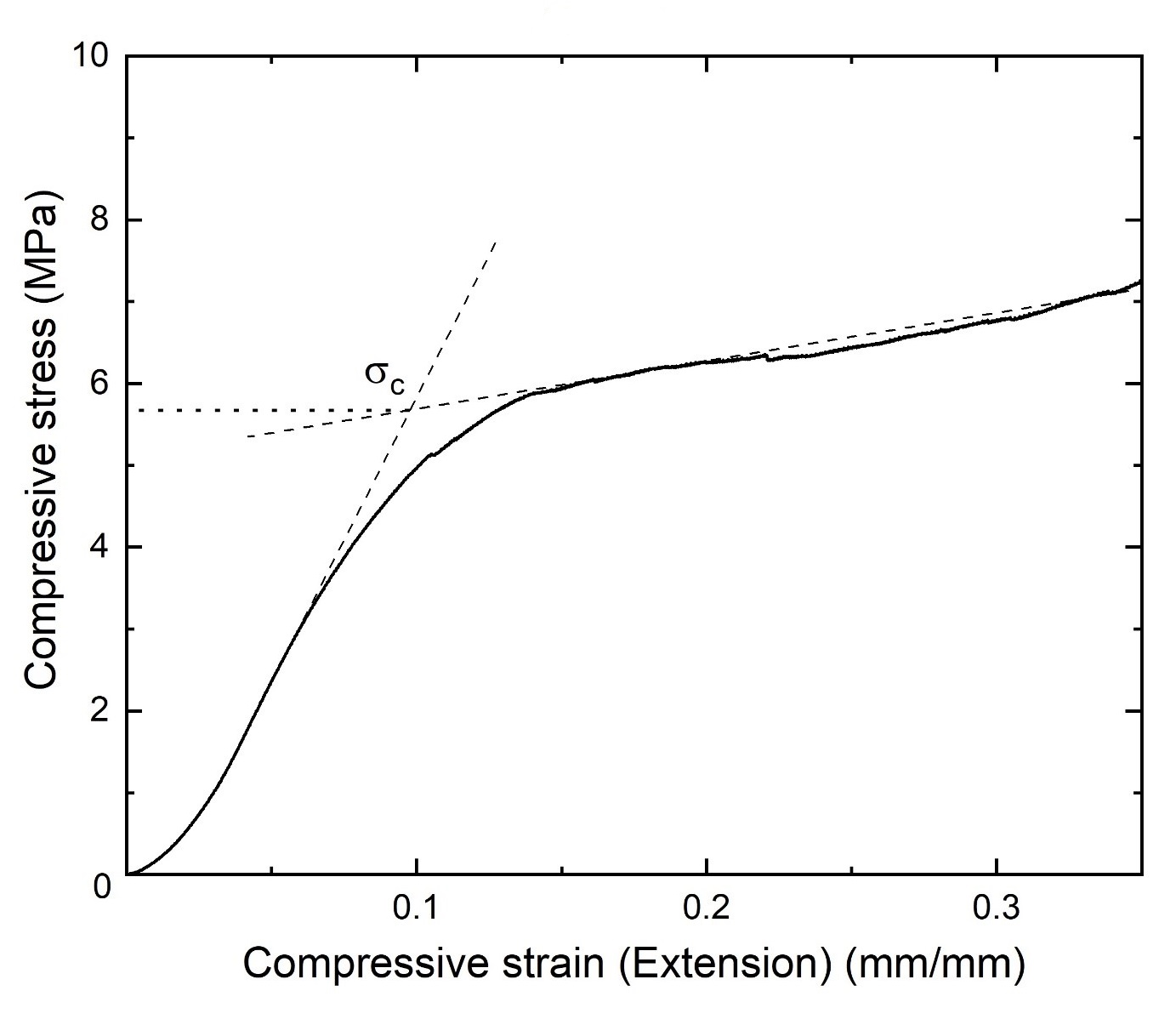


**Fig. S3.** *Illustration of the determination of crush strength (σ_c_)* *for* ***D2*** *sample.*

*
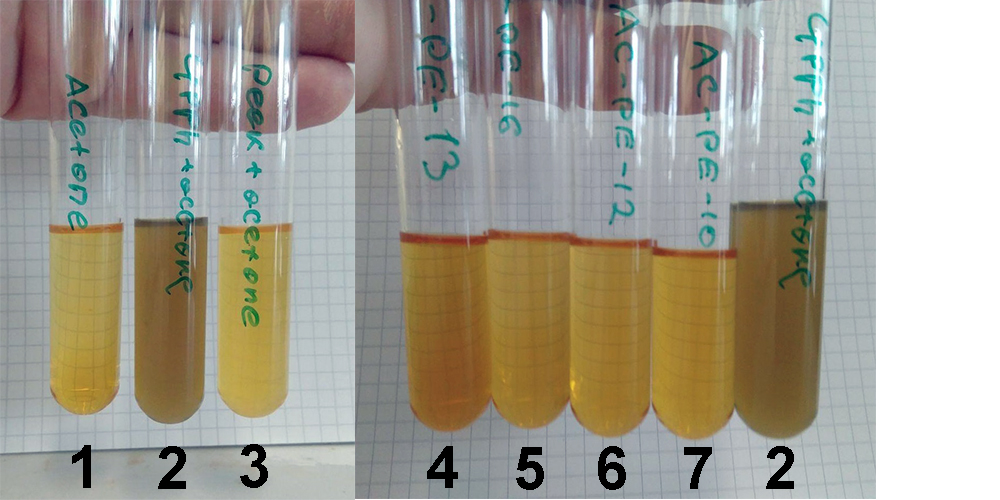
*

**Fig. S4.** *Ferric Chloride Test to define phenol presence.*

*7 samples were prepared for the* *ferric chloride test:*

1. *Pure acetone*
2. *0.5 g 4PPH with 5 g of acetone*
3. *0.5 g of PEEK APC-2 (original PEEK powder) with 5 g of acetone*
4. *0.5 g of ground PE-5-10 sample with 5 g of acetone*
5. *0.5 g of ground PE-5-0.5 sample with 5 g of acetone*
6. *0.5 g of ground PE-20-0.5 sample with 5 g of acetone*
7. *0.5 g of ground PE-20-10 samples with 5 g of acetone*


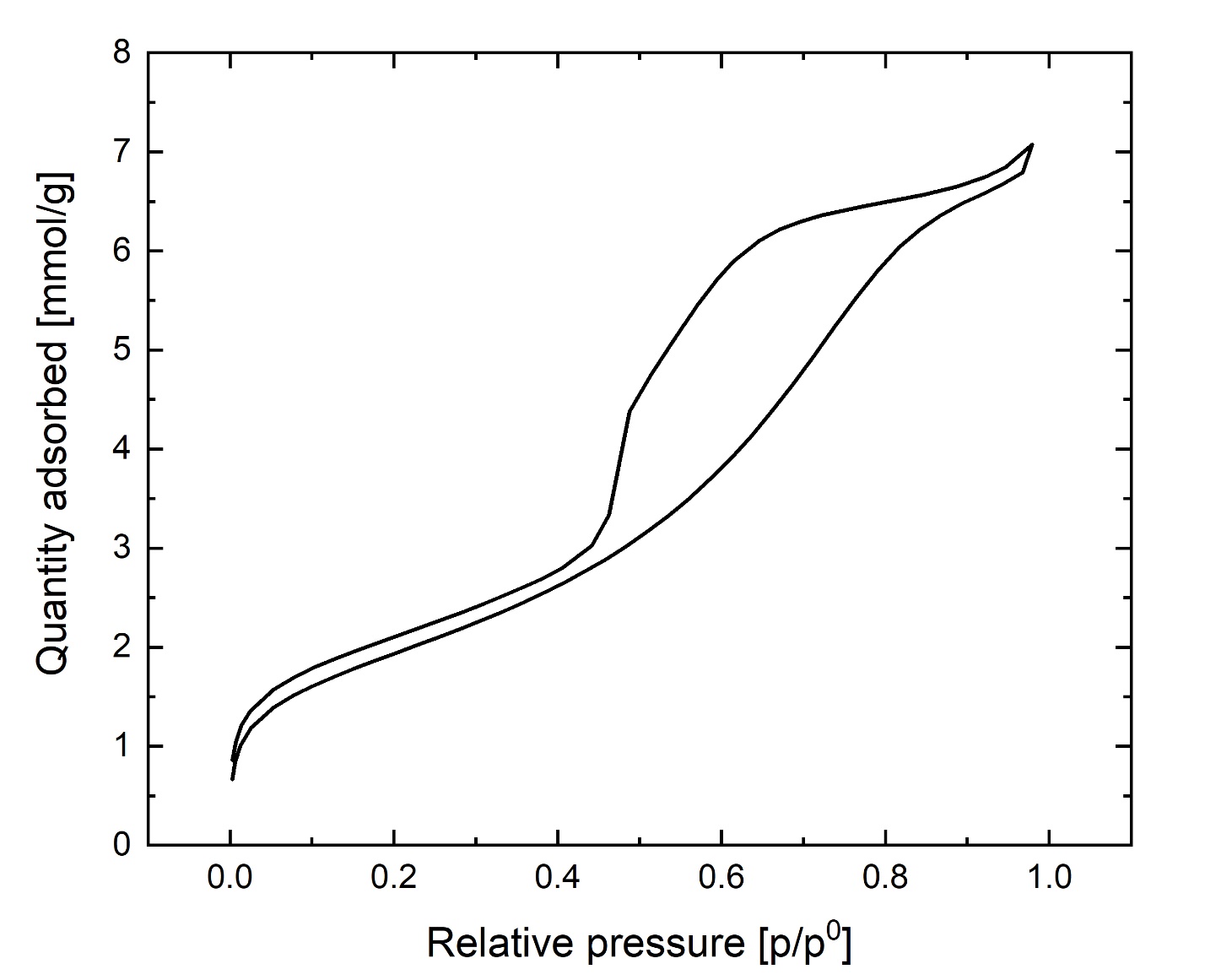


**Fig. S5** *Characteristic nitrogen adsorption isotherm for* ***B2*** *sample.*


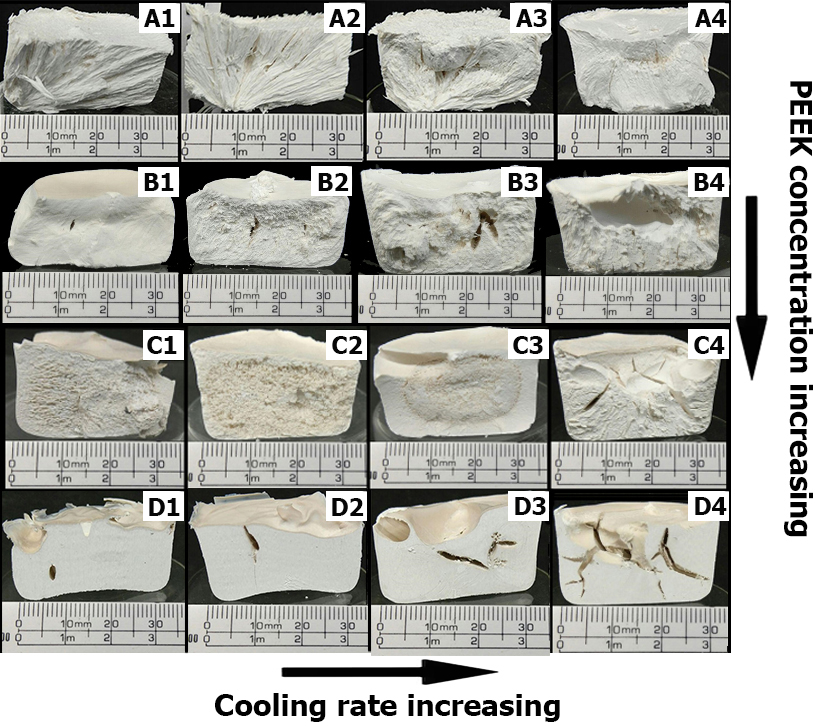


**Fig. S6***. Photographs of PEEK foams after purification, produced by HT-TIPS from 5 wt% (row A), 10 wt% (row B), 15 wt% (row C) 20 wt% (row D) PEEK in 4PPH solutions, at cooling rates β of 1) 0.5 ℃/min, 2) 1 ℃/min, 3) 2 ℃/min, and 4) 10 ℃/min.*


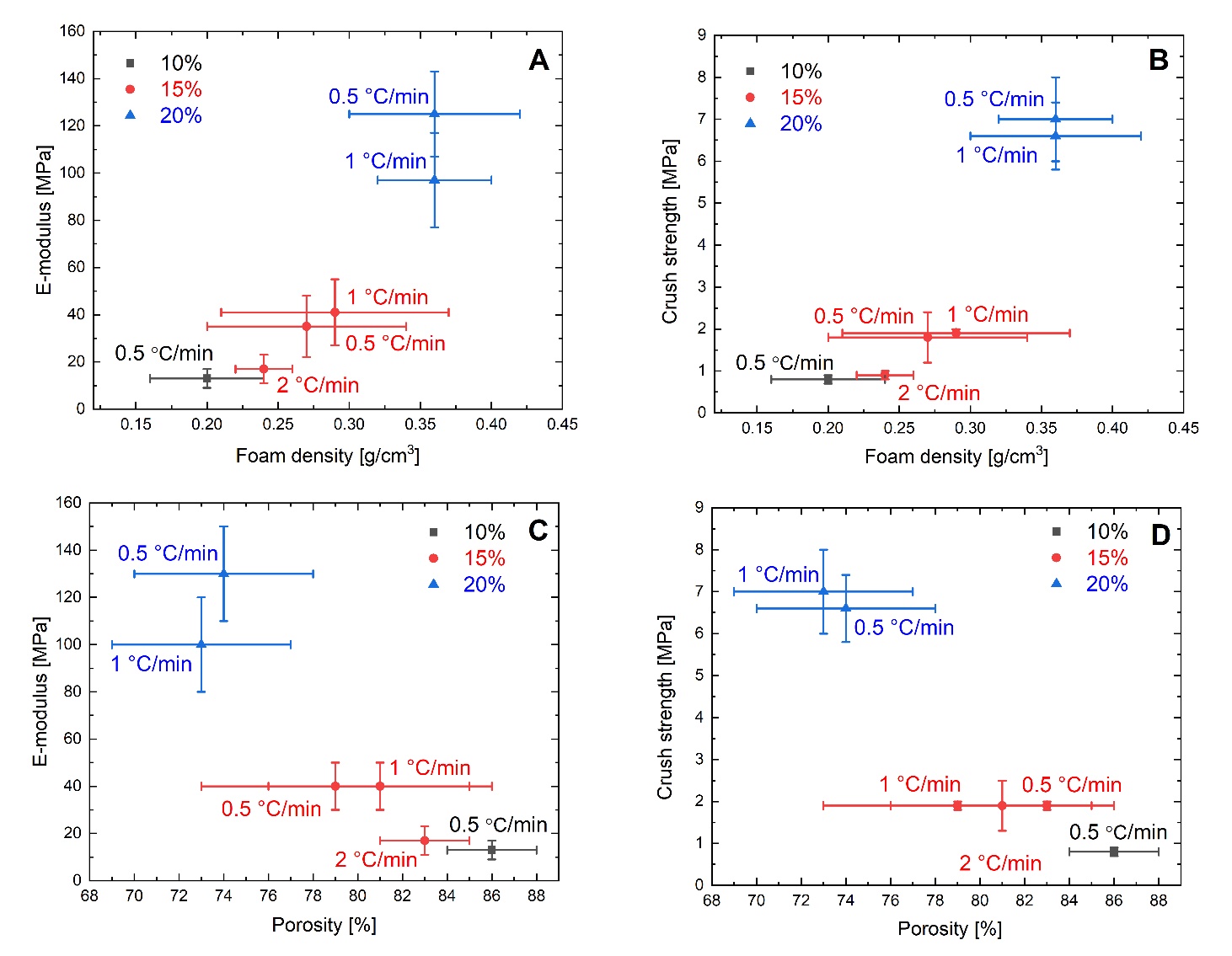


**Fig. S7***. Elastic modulus (left) and crush strength (right) of our PEEK foams at the different cooling rates as a function of foam density (A and B); and as a function of porosity (C and D respectively).*
